# Use of malaria RDTs in various health contexts across sub-Saharan Africa: a systematic review

**DOI:** 10.1186/s12889-017-4398-1

**Published:** 2017-05-18

**Authors:** Matthew R. Boyce, Wendy P. O’Meara

**Affiliations:** 1 Duke Global Health Institute, Durham, NC USA; 2 School of Public Health, Moi University College of Health Sciences, Eldoret, Kenya

**Keywords:** CHW, Drug shop, Health facility, Malaria, *Plasmodium falciparum*, Retail sector, RDT, School, Sub-Saharan Africa

## Abstract

**Background:**

The World Health Organization recommends parasitological confirmation of malaria prior to treatment. Malaria rapid diagnostic tests (RDTs) represent one diagnostic method that is used in a variety of contexts to overcome limitations of other diagnostic techniques. Malaria RDTs increase the availability and feasibility of accurate diagnosis and may result in improved quality of care. Though RDTs are used in a variety of contexts, no studies have compared how well or effectively RDTs are used across these contexts. This review assesses the diagnostic use of RDTs in four different contexts: health facilities, the community, drug shops and schools.

**Methods:**

A comprehensive search of the Pubmed database was conducted to evaluate RDT execution, test accuracy, or adherence to test results in sub-Saharan Africa. Original RDT and *Plasmodium falciparum* focused studies conducted in formal health care facilities, drug shops, schools, or by CHWs between the year 2000 and December 2016 were included. Studies were excluded if they were conducted exclusively in a research laboratory setting, where staff from the study team conducted RDTs, or in settings outside of sub-Saharan Africa.

**Results:**

The literature search identified 757 reports. A total of 52 studies were included in the analysis. Overall, RDTs were performed safely and effectively by community health workers provided they receive proper training. Analogous information was largely absent for formal health care workers. Tests were generally accurate across contexts, except for in drug shops where lower specificities were observed. Adherence to RDT results was higher among drug shop vendors and community health workers, while adherence was more variable among formal health care workers, most notably with negative test results.

**Conclusions:**

Malaria RDTs are generally used well, though compliance with test results is variable – especially in the formal health care sector. If low adherence rates are extrapolated, thousands of patients may be incorrectly diagnosed and receive inappropriate treatment resulting in a low quality of care and unnecessary drug use. Multidisciplinary research should continue to explore determinants of good RDT use, and seek to better understand how to support and sustain the correct use of this diagnostic tool.

**Electronic supplementary material:**

The online version of this article (doi:10.1186/s12889-017-4398-1) contains supplementary material, which is available to authorized users.

## Background

Malaria remains a leading public health problem, causing significant amounts of preventable morbidity and mortality worldwide. This is especially true in sub-Saharan Africa, where an estimated 90% of all malaria deaths occur, and despite recent improvements in diagnosis and treatment, malaria accounts for 10% of under-five mortality and remains a leading cause of death [1].

Previously, malaria case management in sub-Saharan Africa relied on the presumptive treatment of febrile illness with antimalarials, essentially treating suspected cases of malaria with a full course of antimalarial drugs, usually chloroquine or sulphadoxine-pyrimethamine [2, 3]. This practice was endorsed by leading health organizations and widely practiced to reduce malaria-attributable morbidity and mortality in regions where a diagnosis was not feasible [4].

However, malaria treatment policy has changed in recent years as presumptive treatment no longer represents a justifiable approach to malaria case management. In 2010, the World Health Organization (WHO) revised their recommendations to require parasitological confirmation of malaria infection prior to treatment with artemisinin-based combination therapy (ACT), also known as the “test-and-treat” strategy [5]. This change was precipitated by a declining prevalence of malaria in sub-Saharan Africa, likely attributable to the use of modern control interventions, including insecticide-treated nets and indoor residual spraying [6–8]; evidence suggesting that malaria only causes a proportion of all febrile illness in malaria endemic regions [9–11]; concerns surrounding antimalarial drug resistance [12, 13] and improvements in diagnostic technologies. The confirmation of parasitological infection is also important for the management of other febrile illnesses; as disease burden shifts it is necessary to know the infection status of each febrile patient so that they may receive proper care [3].

Three techniques are currently in use for parasitological confirmation of malaria infection: blood smear microscopy, polymerase chain reaction (PCR), and malaria rapid diagnostic tests (RDTs). Microscopic examination of stained blood smears or films represents the oldest laboratory method, and is currently recognized as the gold standard for malaria diagnosis [14–19]. In addition to confirming the presence of malaria parasites, microscopy can also confirm the type of *Plasmodium* species causing infection, as well as score parasite density and differentiate developmental and sexual stages of the parasite [17]. Because of these features, microscopy is especially helpful in not only diagnosing malaria but also assessing the severity of infection [14]. However, microscopy has several notable limitations. Reliable diagnosis requires experienced laboratory technicians with training and technical expertise, high-quality equipment and reagents (e.g. well-maintained microscope, staining reagents, filtered water at the correct pH, etc.), electricity, and it is relatively time-consuming [14–16, 20–22]. Indeed, studies have shown that many health facilities in malaria endemic regions lack the capacity to perform clinical microscopy, making accurate diagnosis unfeasible [15, 23–27].

PCR can identify parasites and infecting species even when parasites are present at very low densities, and some facilities in some developed countries use it as a routine diagnostic method [23, 28–30]. Still, the technology is not able to distinguish between different parasite stages (unless reverse transcriptase PCR is used), or between living and dead parasites, and is subject to many of the same limitations as microscopy, including requiring experienced laboratory technicians, high-quality equipment, specialized reagents maintained at low temperature, and electricity [28, 29, 31]. For these reasons, PCR is generally only used in research settings or to confirm other laboratory findings [18].

Malaria RDTs were developed in the early 1990’s and welcomed as a method to overcome the shortcomings of other laboratory diagnostic techniques, especially in field studies [19, 31–34]. This diagnostic method utilizes chromatography and antigen-antibody recognition to detect parasite antigens including histidine-rich protein-2 (HRP-2) or lactate dehydrogenase (pLDH) [15, 17]. RDTs do not require electricity or specialized equipment and return results in less than 30 min [15, 17].

According to WHO’s recommendations, RDTs should provide diagnostic results at least as accurate as those derived from microscopy under standard field conditions [35]. Test sensitivity poses the most pressing concern, as false-negative results may cause mistreatment of a potentially fatal disease. Subsequently, WHO defines the minimum sensitivity of 95% compared to microscopy, and 100% when parasite density is greater than 100 parasites per μl blood [35]. WHO also defines the minimum specificity of RDTs as 90% when compared to microscopy [35]. A recent systematic review investigating the diagnostic accuracy of RDTs for detecting uncomplicated malaria found that although there was substantial heterogeneity between studies, the sensitivities and specificities of all RDTs tested exceeded WHO’s recommendations, and therefore concluded that RDTs are acceptable to replace microscopy for the diagnosis of malaria [36].

Ultimately, limited access to microscopy for parasitological confirmation of malaria has resulted in the overuse of antimalarials and substandard care for febrile illnesses. There is a compelling need to make malaria diagnosis more available which favors the expanded deployment and use of RDTs. Malaria RDT technology is attractive because it overcomes many of the limitations of other diagnostic techniques. The simplicity of the tests eliminates the need for high levels of technical expertise and allows them to be used by a wide range of personnel including nurses, community health workers (CHWs), teachers, and other laypersons. Perhaps most importantly, the technology has demonstrated acceptable diagnostic sensitivities and specificities [36], showing potential to dramatically reduce the cost of case management and the risk of drug resistance associated with overuse of antimalarial drugs [37–39].

The expanded use of RDTs in contexts where microscopy is not feasible has been researched extensively in several sub-Saharan African countries. However, to our knowledge, no studies have compared how well or effectively RDTs are used across this broad range of contexts. Thus, comparing the use of RDTs in different environments represents a current gap in knowledge regarding the treatment and diagnosis of suspected malaria cases. Here we assess the diagnostic use of RDTs in four different contexts: health facilities, the community, drug shops and schools, examining how well the test is executed and how RDTs impact the prescription of antimalarial medication. We then discuss the quality of RDT use across these contexts and implications for malaria case management.

## Methods

### Database search & screening

A systematic search and review was conducted following the Preferred Reporting Items for Systematic Reviews and Meta-Analyses (PRISMA) guidelines. Synonyms for ‘malaria,’ ‘RDT,’ ‘hospital,’ ‘CHW,’ and ‘drug shop,’ were combined to identify all relevant studies. An additional file provides the complete search syntax (see Additional file 1). The Pubmed database was used for the search. Web of Science was used to search for relevant studies that were in references and citing articles. The search was limited to publications from 2000 onwards, and the last search was conducted in December 2016. There was no restriction placed on outcome or study design. To limit bias in geographic scope no restriction was placed on the language of publication. Two authors (MRB and WPO) collaborated on screening article titles and abstracts for inclusion and exclusion criteria. A second screening was performed on full-text articles. Discrepant results were discussed between the two authors until a unanimous decision was reached.

### Selection of studies

The criteria for inclusion were: original RDT focused studies conducted in formal health care facilities, drug shops, schools, or by CHWs; studies primarily investigating *Plasmodium falciparum* malaria; studies evaluating one or more of the following steps: test performance by health care providers of interest, accuracy of RDT results performed by health care providers of interest, test interpretation, and adherence to test results by health care providers of interest. *P. falciparum* was the focus of this review because it is the most prevalent on the African continent and results in the highest mortality rates. This study considered the formal health care sector to be environments that included public and private hospitals, health centers, clinics, and dispensaries. The retail sector included private drug shops and pharmacies staffed by licensed pharmacists or informally trained shop vendors. Schools included primary or secondary schools. Use by CHWs was defined as RDT use by non-professional health care workers who provided services at the community level. Execution of tests included the performance of all RDT procedure steps from start to finish and is synonymous with test performance or handling. Execution may be described as ‘safe’ or ‘correct’ and it is possible to test the ‘quality of execution.’ Test safety is a subset of these steps and referred to procedure steps that related to the safety of the health worker or patient. Test accuracy referred to the sensitivity and specificity of RDT results relative to a gold-standard measure. Interpretation referred to correctly reading test results (e.g., not interpreting a positive result as negative or invalid). Adherence referred to complying with treatment guidelines based on RDT results. This study defined appropriate treatment as prescribing antimalarials to RDT-positive patients and not prescribing antimalarials to RDT-negative patients; inappropriate treatment was defined as prescribing antimalarials to RDT-negative patients. An additional file shows these data in more detail (see Additional file 2).

Exclusion criteria were: studies conducted exclusively in a research laboratory setting, studies where staff from the research team conducted RDTs, and studies outside of sub-Saharan Africa. Studies conducted in a research setting or where study staff conducted RDTs were excluded because this systematic review sought to investigate the use of RDTs in clinical settings by health care workers. Sub-Saharan Africa was the location of interest because of the well-documented burden of *P. falciparum* [1].

## Results

**Fig. 1:**
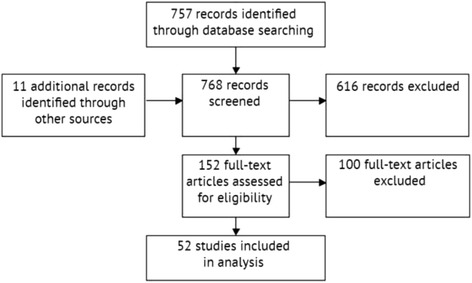
Study selection diagram

A total of 757 titles published from 2000 to present were identified from the database search. Full texts of 152 studies were retrieved, of which 100 were not included due to the previously stated exclusion criteria. One study written in French was identified and reviewed by one of the authors (WPO), though it was excluded as it did not meet the inclusion criteria. Upon completion of the screening process, 41 studies met the inclusion criteria. Eleven additional studies were identified from the references of other studies and included in the review for a total of 52 included studies (Fig. 1). No systematic patterns were observed in these studies, allowing for confidence in the robustness of the search strategy. It is thought that the 11 additional studies may have been excluded because of minor discrepancies between the search strategy and study titles.

### Study characteristics

Geographical restrictions required studies to be conducted in sub-Saharan Africa. Thirteen studies were conducted in Tanzania [16, 40–51], eight in Uganda [52–59], six in Zambia [22, 60–64], three in Burkina Faso [37, 65, 66], three in Ethiopia [67–69], three in Ghana [70–72], two in Kenya [73, 74], two in Malawi [75, 76], two in Mali [77, 78], two in Nigeria [19, 79], one in Cameroon [80], one in the Democratic Republic of Congo [81], one in Madagascar [82], one in Mozambique [83], one in Senegal [84], one in Sierra Leone [15], one in South Africa [85], and one in multi-country settings [86].

The context restrictions allowed for studies to be conducted in four distinct contexts – the formal health care sector, the retail sector, schools, and in the community by CHWs. Thirty of the studies reviewed were conducted in the formal health care sector [15, 19, 22, 37, 40–46, 48–51, 53, 55, 56, 65, 67, 68, 71–75, 77, 80, 83, 85]. Nearly all in public or government run hospitals, though a number were conducted in other facilities such as peripheral health centers, outpatient clinics, dispensaries, and community clinics. Of these studies, one contained data regarding RDT performance [85], 20 contained data regarding sensitivity and specificity [15, 43–46, 48–51, 55, 56, 65, 67, 68, 71–73, 75, 77, 85], and 14 contained data regarding adherence to results and antimalarial prescription [19, 22, 37, 40–42, 46, 49, 50, 53, 74, 75, 80, 83]. Five studies that were included examined the use of RDTs in the retail sector of sub-Saharan Africa [52, 54, 57, 70, 79]. Across these studies, one contained data regarding the execution of RDTs [70], two contained data regarding sensitivity and specificity [57, 70], and all five contained data regarding adherence to RDT results. Sixteen studies examined the use of RDTs by CHWs at the community level [16, 47, 58–64, 66, 69, 78, 81, 82, 84, 86]. Six of these studies contained data regarding RDT execution [47, 61, 62, 64, 69, 81], six contained data regarding sensitivity and specificity [16, 47, 59, 66, 78, 82], and seven contained data regarding adherence to RDT results [47, 58, 60, 62, 63, 84, 86]. Lastly, one study was conducted in a school that investigated RDT execution [76].

Both experimental and descriptive studies were eligible to be included in this review. Of the 52 studies included, two were randomized controlled trials [37, 49], 12 were cluster randomized trials [41, 53, 54, 57, 59, 62, 70, 73, 74, 76, 80, 86], one was a randomized cross-over trial [47], two were quasi-experimental studies utilizing a pre−/post-assessment design [40, 52], and 35 were observational studies [15, 16, 19, 22, 42–46, 48, 50, 51, 55, 56, 58, 60, 61, 63–69, 71, 72, 75, 77–79, 81–85]. Several the observational studies were further classified as cross-sectional studies [16, 19, 48, 50, 58, 68, 69, 72, 75], longitudinal studies [16, 56, 61, 63], and cohort studies [51, 81]. An additional file shows study characteristics in more detail (see Additional file 2).

The heterogeneity of study design, context, and outcomes made quantitative methods, including meta-analysis, unfeasible and inappropriate. Studies are therefore described and reported in the following narrative.

### Formal health care sector

One of the studies directly investigated the execution of RDTs in the formal health care setting [85]. This study demonstrated that nurses and nursing assistants were not proficient in the safe execution of RDTs, only correctly performing between 8% and 84% of the 14 procedure steps. The reporting of these data made it impossible to determine if any health worker completed all 14 steps correctly, but eight of the 14 steps were completed by at least 70% of the workers. Of importance, less than 80% of the health workers put on a new pair of gloves, checked the test expiration date, used a sterile lancet to prick the patient’s finger, properly disposed of the used lancet, dispensed the buffer correctly, waited the correct amount of time to read results, and correctly interpreted results. One additional study that included interviews with health care workers also presented results about self-reported RDT performance [19]. The researchers found that of the 32 nurses interviewed, 6.3% (*n* = 2) practiced unsafe handling or disposal of sharps, 31.3% (*n* = 10) had difficulty drawing and collecting blood samples, 25% (*n* = 8) had difficulty transferring blood to the test device, and 15.6% (*n* = 5) read results too soon (defined before 15 min had elapsed).

Sensitivity and specificity of RDT results from these clinical settings were variable (Table 1). Most the studies used microscopy as the gold-standard comparator and demonstrated sensitivities ranging from 64.8% [43] to 100% [71], whereas specificities ranged from 39% [75] to 99.7% [50]. However, most studies reported sensitivities of at least 90% and specificities of at least 80%. Two studies used PCR as the gold-standard and reported similar sensitivities and specificities values [48, 50].

**Table 1:** Reported RDT sensitivity and specificity data for included studies

Author	Year	Sensitivity (95% CI)	Specificity (95% CI)
*Formal Health Care Sector*			
Ashton	2010	85.6%	92.4–92.7%
Baiden	2012	100%	73.0% (67–78)
Chinkhumba	2010	90–97%	39–68%
de Oliveira	2009	91.7 (80.8–100.0)	96.7 (92.8–100.0)
Diarra	2012	89.6% (88.1–90.9)	81.1% (78.8–83.2)
Gerstl^a^	2010	99.4% (96.8–100.0)	96.0% (91.9–98.4)
Gerstl^b^	2010	98.8% (95.8–99.8)	74.7% (67.6–81.0)
Guthmann	2002	97%	88%
Hopkins^a^	2007	85%	99.8%
Hopkins^b^	2007	92%	93%
McMorrow	2008	64.8%	87.8%
McMorrow	2010	90.7%	73.5%
Moonasar	2009	85%	96%
Morankar	2011	93%	99.4%
Msellem	2009	94%	88%
Mtove	2011	97.5% (96.9–98.0)	65.3% (63.8–66.9)
Nicastri^*p*^	2009	69.2%	100%
Osei-Kwakye	2013	97.7% (95.8–99.0)	58.1% (53.8–62.3)
Ouattara	2011	97.2%	95.4%
Shakely	2013	78.6% (70.8–85.1)	99.7% (99.5–99.9)
Shakely^*p*^	2013	76.5% (69.0–83.9)	99.9% (99.7–100)
Shekalaghe	2013	94.7% (89.8–97.3)	95.6% (94.2–96.6)
*Retail Sector*			
Ansah	2015	98–100%	30–98%
Mbonye	2015	91.7%	63.1%
*Community Health Workers*			
Ishengoma^*c*^	2011	88.6%	88.2%
Ishengoma^*d*^	2011	63.4%	94.3%
Mubi	2011	85.3%	59.8%
Ndyomugyenyi^e^	2016	72.1%	83.3%
Ndyomugyenyi^f^	2016	20.8%	98.1%
Ratsimbasoa^*e*^	2012	90.2% (81.7–95.7)	87.2% (78.3–93.4)
Ratsimbasoa^*f*^	2012	93.7% (69.8–99.4)	83.3% (35.9–99.6)
Tiono	2013	97.9% (96.3–98.8)	53.4% (49.1–57.7)
Willcox	2009	82.9% (78.0–87.1)	78.9% (63.9–89.7)

Most studies used microscopy as a gold standard, those that used PCR are denoted with a *p*. Two studies [15, 56] used two different types of RDTs and calculated separate sensitivities and specificities for each; RDT sensitivity and specificity using a pLDH RDT is denoted with an *a*; RDT sensitivity and specificity using a HRP-2 RDT is denoted with a *b*. Another study [16] included sensitivity and specificity data from a cross-sectional study nested in a larger longitudinal study; RDT sensitivities and specificities from the longitudinal study are denoted with an *c*; RDT sensitivities and specificities from the cross-sectional study are denoted with a *d*. Two studies [59, 82] included sensitivity and specificity data from different transmission seasons; RDT sensitivities and specificities from higher-transmission seasons are denoted with an *e*; RDT sensitivities and specificities from lower-transmission seasons are denoted with a *f*. Ansah et al. reported sensitivities and specificities of individual drug shops, but not an overall value for either measure [70]. As such, a range of sensitivities and specificities is reported. Confidence intervals were included if reported in the original study

Fourteen of the 30 studies conducted in this context investigated the appropriateness of treatment following RDT diagnosis (Table 2). Appropriate treatment ranged from 54.4% [49] to 99.9% [50]. Four of these studies [19, 40, 50, 53] showed that all patients who tested positive for malaria using an RDT received appropriate antimalarial medication; several other studies reported similar results, with greater than 95% of all RDT-positive patients receiving antimalarials [22, 37, 42, 49, 75, 83]. Only three studies showed less than 90% of RDT-positive patients receiving appropriate antimalarial medication [41, 48, 80].

**Table 2:** Appropriate treatment overall, RDT-positive and RDT-negative results

Author	Year	Appropriate Treatment (%)	Positives Treated (%)	Negatives Not Treated (%)
*Formal Health Care Sector*				
Bastiaens	2011	90.4%	100.0%	90.0%
Batwala	2011	88.5%	100.0%	76.6%
Bisoffi	2009	60.7%	97.7%	19.0%
Bottieau	2013	93.4%	95.1%	92.8%
Chinkhumba	2010	86.9%	98.0%	57.9%
Cundill	2015	91.4%	80.3%	95.1%
Hamer	2007	78.7%	96.6%	64.5%
Masanja	2010	95.9%	95.8%	96.0%
Mbacham ^*a*^	2014	56.1%	72.1%	48.1%
Mbacham ^*b*^	2014	70.8%	72.9%	69.4%
Nicastri	2009	66.4%	55.6%	67.0%
Reyburn	2007	54.4%	98.9%	46.3%
Shakely	2013	99.9%	100.0%	99.9%
Skarbinski	2009	88.0%	92.9%	87.2%
Uzochukwu	2011	60.0%	100.0%	25.9%
*Retail Sector*				
Ansah	2015	97.7%	99.5%	93.8%
Awor	2015	91.1%	93.5%	82.8%
Cohen	2015	80.0%	83.3%	56.3%
Ikwuobe	2013	55.4%	100.0%	48.4%
Mbonye	2015	98.8%	99.0%	98.5%
*Community Health Workers*				
Chanda	2011	98.4%	98.4%	98.4%
Hamainza	2014	83.2%	61.6%	98.0%
Hamer	2012	99.3%	98.5%	99.6%
Mubi	2011	96.8%	99.7%	93.9%
Mukanga	2011	96.7%	96.5%	97.5%
Mukanga	2012	99.1%	99.9%	95.1%
Thiam	2012	-	96.6%	-

*‘a’* denotes appropriate treatment for clinicians in the basic intervention group of the Mbacham study; *‘b’* denotes appropriate treatment for clinicians in the enhanced intervention group of the Mbacham study [80]. Thiam and colleagues did not report the number of negatives not treated, making the calculation of the total amount of appropriate treatment inappropriate [84]

**Fig. 2:**
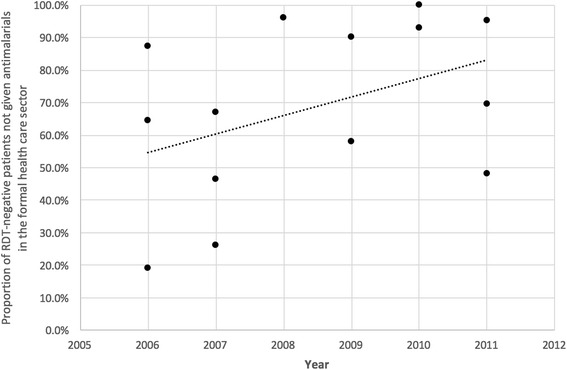
Temporal trend in the proportion of RDT-negative patients not treated given antimalarials in studies conducted in the formal health care sector

The results for inappropriate treatment were more variable. Studies showed that the percentages of patients receiving antimalarials inappropriately ranged from 0.1% [50] to 81% [37], although most studies reported significant percentages of patients receiving inappropriate antimalarial treatment. Ten of the 14 studies reported at least 10% of RDT-negative patients receiving antimalarial drugs [19, 22, 37, 40, 48, 49, 53, 74, 75, 80]. A temporal trend was observed where the proportion of RDT-negative patients not receiving antimalarials increased over time (Fig. 2).

### Retail sector

The studies conducted in the retail sector most often focused on evaluating the impact of RDTs on antimalarial drug prescription, drug shop vendor (DSV) adherence to RDT results, referral practices, and overall appropriateness of treatment. One study produced results with data about the execution of RDTs [70]. Ansah and colleagues directly observed testing in the shops and reported that medicine retailers followed instructions. The vendors performed well across all 18 safety indicators assessed in the study, ranging from 87.2% to 100% of steps completed correctly [70].

Results for the accuracy of RDTs in the retail sector suggested that sensitivity was high, ranging from 91.7% [57] to 100% [70] when using microscopy as a gold standard (Table 1). Specificity results were much more variable. One study reported that in the 28 shops using RDTs, most had specificities between 73% and 98% [70]. However, three of the shops reported low specificities of 30%, 31%, and 52% [70]. The other study yielded similar results where researchers re-read tests that were stored and found a low specificity of 63.1%, with a high number of false-positive tests [57]. In this study, over one-third of RDT-positive clients were parasite negative by expert microscopy [57].

Studies investigating prescription of antimalarials following an RDT showed that DSVs generally provided treatment in accordance with the RDT results (Table 2). Results from these five studies show that appropriate treatment based on RDT result was as low as 55.4% in a study conducted in Nigeria [79], to 98.8% [57]. The remaining studies all showed that drug shops supplied appropriate treatment to at least 80% of tested patients. It should also be noted that the low end of this range was produced by a study involving only one intervention-drug shop. Furthermore, one of these studies was conducted in Ghana where the sale of antibiotics over the counter is not permitted by law [70]. In other contexts, where antibiotics are readily available over-the-counter, a shift to the prescription of antibiotics instead of antimalarials is probable.

Similar to the formal health care sector, results from these studies showed that inappropriate treatment of RDT-negative patients with antimalarials was more variable than appropriate treatment of RDT-positive clients. Two studies suggested that over 90% of RDT-negative individuals did not receive antimalarials [57, 70], one study showed that least 10% of RDT-negative patients receiving antimalarial drugs [52] while the other two studies showed that approximately half of all RDT-negative individuals inappropriately received antimalarials [54, 79].

### Community health workers

Sixteen studies examined the use of RDTs by CHWs. The studies included were heterogeneous in study design and highly variable in size, ranging from eight CHWs to 408 CHWs. One of the included studies sought to investigate whether CHWs could prepare and interpret RDTs accurately and safely using manufacturer’s instructions alone, or if additional job aids improved performance [64]. These researchers found that CHWs completed 57% of the 16 steps correctly using only the manufacturer’s instructions, 80% with a job-aid, and 90% with a job-aid plus training. CHWs using only the manufacturer instructions frequently documented the test incorrectly, forgot to check the test expiry date, frequently did not use gloves, and inappropriately discarded used materials. These errors in use, though still committed by CHWs receiving additional training, were much less frequent. This finding was supported by other researchers who evaluated how well CHWs performed RDTs, and found that CHWs who received extended training and supervision significantly out-performed their counterparts with 39.1% (34/87) of these CHWs completing greater than 80% of the steps correctly compared to 21.0% (16/76) of CHWs who did not receive this additional training [69]. Subsequent studies used trained observers to assess CHW performance, with attention to blood- and patient-safety concerns [47, 62, 81]. These studies showed that between 76% and 100% of CHWs safely completed critical steps in the RDT procedure based on structured, study specific observation checklists, and all reported that CHWs demonstrated the ability to perform RDTs safely and effectively. Additional work also concluded that adequately trained and appropriately resourced CHWs can perform and interpret RDTs at an acceptable level, but did not explicitly investigate the safety of RDT handling by CHWs [66].

In a longitudinal study involving 61 CHWs, Counihan and colleagues found that median CHW performance remained steady or improved over time for critical steps, non-critical steps, and RDT interpretation, and that the median percentage of critical RDT steps performed correctly rose from 87.5% at three months to 100% at six and 12 months [61]. The only measure that did not improve involved the ability to correctly interpret RDT results. Using photographs of 10 different RDT results, CHWs correctly identified 96.5% of positive tests at three months and 98.3% at six months, but only 90.5% at 12 months. Similarly, CHWs improved from correctly identifying 94.3% of negative results at three months to 97.9% at six months, but regressed to 94.7% at 12 months. Community health workers’ interpretation of invalid test results improved from 90.2% correct at three months to 96.7% at six months and 96.5% at 12 months. The same pattern held for the interpretation of faint-positive test lines as positive, which improved from 89.7% at three months to 96.7% at six months, but then declined to 76.7% at 12 months. Taken together, these results indicate acceptable execution of RDTs by CHWs, which is enhanced by training, regular supervision, and feedback, and that these skills are maintained over time, presumably through practice.

Six studies included data on the sensitivity and specificity of RDTs when used by CHWs, all of which used microscopy as the gold-standard. Sensitivities of the tests ranged from 20.8% [59] to 97.9% [66], and specificities from 53.4% [66] to 98.1% [59] (Table 1). Five of the six reported sensitivities over 80%, while four of the six reported specificities greater than 75%. The lowest sensitivity was recorded in a trial conducted during a low-transmission season, though the researchers also reported a higher sensitivity in a season with higher rates of malaria transmission [59].

Seven of the 16 studies involving CHWs investigated the appropriateness of treatment and showed that CHWs display high levels of adherence to treatment guidelines (Table 2). All studies showed CHWs providing appropriate treatment at least 80% of the time, with a range of 83.2% [63] to 99.3% [62]. Only one study showed fewer than 90% of RDT-positive patients not receiving treatment [63], and the other six showed that greater than 95% of RDT-positive patients received an antimalarial [47, 58, 60, 62, 84, 86]. Studies showed that CHWs rarely provided inappropriate treatment of RDT-negative patients with antimalarials. Only one study showed greater than 5% of RDT-negative patients received an antimalarial [47].

### Schools

One study was conducted by Witek-McManus and colleagues in a primary school [76]. This evaluation involved 107 teachers in southern Malawi and assessed whether trained teachers executed RDTs correctly, provided appropriate treatment with ACT, and whether this competence was retained up to seven months post-training. Following the final training, teachers completed an average of 93% (19.5/21) of RDT steps correctly. Except for checking the expiry date of the RDT, each step was correctly carried out by ≥80% of teachers. The results of this study showed that teachers could safely perform RDTs and accurately interpret results. Furthermore, this competence was retained over the seven month timeframe, though some procedural errors did arise over the seven months that have implications for monitoring RDT performance by teachers and future studies involving similar populations.

## Discussion

This review examines how well malaria RDTs are executed and how their use impacts the prescription of antimalarial medication in different contexts – the formal health care sector, in the community, in the retail sector, and in schools. To the authors’ knowledge, this is the first study comparing multiple aspects of the use of RDTs (execution, accuracy, and adherence) in these distinct contexts across sub-Saharan Africa.

### Formal health care sector

Rapid diagnostic tests are becoming increasingly common in the formal health care sector. Although microscopy has long been the standard of care in clinical settings, it requires technical expertise, a functional microscope, electricity, and specialized reagents [14] which may not be available in a large fraction of lower-level facilities. Furthermore, even when microscopy is available, time and human resource constraints may preclude testing of every suspected malaria fever when patient volume is high. Because of these constraints, there is increasing investment in scaling up the use of RDTs to expand malaria testing coverage.

The results of this review highlight a lack of data pertaining to the execution of RDTs in the formal health care sector. The limited data available in the peer-reviewed literature suggest that RDTs may not be performed consistently in a safe or effective manner [85]. The literature is more robust as it relates to sensitivity and specificity, and the effect of RDT use on antimalarial prescription practices. Most studies reported sensitivities of at least 90% and specificities of at least 80% for RDTs executed by health workers during routine care. Despite adequate accuracy, adherence to RDT results, particularly negative results, is sub-optimal. More than half of the studies reported that at least 30% of RDT-negative clients received an antimalarial. Researchers have emphasized a level of skepticism towards RDT results among both patients and health care workers, due to a high rate of negative RDT results, as compared with the expectations of both patients and providers [87, 88]. This likely contributes to the inappropriate treatment of RDT-negative individuals. Distrust is further increased when RDT-negative patients later test positive by microscopy, which may be attributed to rare occurrences of other malaria species, HRP-2 deletions, or excess parasite antigens (known as the ‘prozone effect’) [89], but more likely, are simply due to poor clinical microscopy that results in an incorrect diagnosis. Other work has shown that formal health workers require experience with positive RDT results before they have high levels of confidence in the results produced by RDT [49, 90]. As health workers gain confidence in test results, an increase in adherence to treatment guidelines should be expected. This offers one plausible explanation for the increase in the proportion of RDT-negative patients not given antimalarials in the formal health care sector over time.

Patients’ expectations can impact health care workers’ adherence to RDT results. Patients may expect to be treated for malaria, regardless of test results [87]. Most health care workers acknowledged that this issue was prevalent and problematic, articulating pressures to inappropriately antimalarials to RDT-negative patients. This pressure may be amplified when health care workers lack specific guidelines or tools to aid in the diagnosis and treatment of non-malarial febrile illness, especially when presented with a seriously ill patient [88]. Thus, there is a need for specific guidelines for treating febrile illness, especially with regard as to how health workers should proceed in the event of negative test results.

### Retail sector

Though formal health care facilities may see the majority of malaria cases, provide higher quality care, and provide a wider range of services, long wait and travel times often result in patients seeking care elsewhere. In many cases, retail outlets represent the first and only place of treatment, owing to their accessibility, affordability, and orientation towards satisfying consumer needs [91–95]. Though few of these outlets are staffed by licensed pharmacists – more often staffed by informally trained vendors [96, 97] – an estimated 50% of all antimalarial medication is distributed through drug shops [91, 92]. As such, it is important to consider the use of RDTs in the retail sector, especially their potential role in improving the quality of care and targeting antimalarials to confirmed malaria cases.

Despite their importance in fever management, we identified only five studies of RDTs in retail medicine outlets. Furthermore, only one of these examined the execution of RDTs and two reported sensitivity and specificity data. The results suggest that RDTs are safely executed by DSVs, and that RDTs are highly sensitive in this context, but lack specificity. Low specificities observed in the retail sector may stem from the false reporting of positive RDTs to justify the sale of antimalarials to clients [70]. High rates of false positives may ultimately undermine confidence in RDT results and diminish the perceived importance of testing before taking antimalarials. Reducing these situations is crucial to the wide-spread use of RDTs as they work to undermine confidence in RDT results and the test-and-treat guidelines, ultimately challenging the current treatment recommendations [71]. This raises the concern about how to monitor RDT use in a sector that is often poorly regulated.

DSVs generally provided appropriate treatment following RDT use, indicating that RDTs have the potential improve the quality of care and reduce overuse of antimalarial drugs in this context. In four of the five studies, 80% or greater of the patients received appropriate treatment, while a single study involving only one intervention shop reported substantially lower rates of appropriate treatment [79].

There are several additional considerations that must be acknowledged within this context. For DSVs, a primary consideration is whether RDTs are viewed as a valuable retail product. As opposed to public health facilities, drug shops, and pharmacies are established for profit. As a result, asking DSVs to restrict the sale of ACT exclusively to RDT-positive clients may present a conflict of interest [70, 79]. Furthermore, the provision of RDTs in the retail sector potentially raises a new set of issues and challenges such as the management of severe illnesses, appropriate treatment of RDT-negative fever cases, and referral of patients. Work has shown that within drug shops there is limited awareness of current treatment and diagnosis guidelines, a lack of training in national guidelines, a lack of reference materials, limited record keeping, and weak linkages with the formal health care system [98].

From a patient perspective, it is important to recognize that patients come to the retail sector to purchase a product (i.e., antimalarial medication), as opposed to seeking a diagnosis. Thus, patients have different expectations for the care they will receive in this context [70]. One study suggested that patients may be more inclined to purchase an antimalarial medication, despite RDT-negative status, if it had been recommended by a health professional compared to self-referral, if there was a positive malaria lab test prior to presenting to the drug shop, if the patient had experienced fever in the last 48 hours, and if the patient’s educational level was primary school education or less [79]. Another consideration is whether patients trust the RDT results from the retail sector. Research has demonstrated that when patients were asked which settings could be trusted to provide RDTs, the majority of individuals indicated formal health care facilities, while diagnostic laboratories and pharmacies received lower marks [99].

Lastly, from a broader policy perspective, it is critical to note that a positive RDT result does not necessarily translate into the purchase of ACT. Individuals who frequent drug shops are more likely to purchase substandard, non-ACT antimalarials [99]. DSVs continue to sell non-ACT antimalarials, perhaps due to client demand. Several studies have shown that retail outlets tend to respond to customer demand, and that DSVs may avoid referring patients for confirmatory blood tests because they fear losing business due to added inconvenience, cost, or both [100]. As a result, client preferences for presumptive treatment and non-recommended drugs may be major factors in seeking care in the retail sector, both of which contradict the intent of using RDTs in this context [101].

### Community health workers

Many individuals with suspected malaria in sub-Saharan Africa die without contact with formal medical services, especially in rural and other medically underserved areas [102, 103]. For this reason, the World Health Organization has recommended home-based management of malaria (HMM) by CHWs [104] to increase prompt malaria diagnosis and treatment and decrease malaria-related mortality. Through this strategy, lay health persons receive education about the treatment of malaria, the administration of antimalarial drugs, as well as the recognition of severe illness, and are provided medications to distribute to patients [104, 105]. Though this approach initially relied upon presumptive treatment, it now abides by the more recent test-and-treat recommendations, relying heavily on the integration of RDTs into HMM programs [106, 107].

The services provided in the community by CHWs play an important role in increasing health care coverage and may also reduce workload in the formal sector [108–110]. Therefore, it is important to understand how RDTs are used by CHWs and how their use impacts treatment. The current literature demonstrates that RDTs are performed safely when proper training is provided, that RDTs are generally highly sensitive and specific when executed by CHWs, and that CHWs display high levels of adherence to treatment guidelines. Several of the included studies showed that supplying CHWs with additional training and job aids significantly improved their performance of RDT procedures [62, 64, 69]. Still, while these results may give cause for optimism, the literature is not as extensive as that in the formal health care sector and some concerns remain. These apprehensions arise from uncertainty in the competence of CHWs due to their education levels and novel, additional responsibilities [61, 111]. Most frequent these concerns center around test monitoring as it relates to blood safety, ability to interpret test results correctly, and inappropriate prescription of antimalarial drugs [61, 111, 112].

Storage conditions represent another crucial aspect that must be given consideration when deploying RDTs at the community level, as their accuracy is directly linked to their storage conditions [16]. One study that examined the storage and long-term stability of RDTs in the community found that they were kept under conditions sufficient for high performance and long-term stability [82], however, strategies for monitoring and enforcing adequate storage conditions in large-scale implementation programs are needed.

### Schools

With malaria disproportionately affecting children and increasing levels of enrollment in primary schools throughout sub-Saharan Africa, a practical opportunity to improve timely diagnosis and treatment with antimalarials in schools has been recognized. Building upon the observed successes of training lay-persons as CHWs some researchers have proposed to do the same with school teachers [76]. Though only one study was conducted in this context, it demonstrated teachers could execute RDTs well and accurately interpret results. Additional research is needed to determine how RDT use in schools may impact the prescription of antimalarials.

There are some obvious limitations and additional considerations to this approach. On the one hand, children suffering from malaria may not attend school and would not benefit from this strategy. Conversely, parents may decide to send their febrile children to school knowing they can be tested and treated at school, though this may raise concerns pertaining to the well-being of the child. Furthermore, in the case of non-malarial febrile-illnesses (i.e., viral), the presence of a sick child could place other children at risk. Additionally, taking time out from regular duties of teachers to care for sick children could prove problematic; the added responsibility of performing an RDT may result in teachers underperforming in more routine classroom responsibilities. Lastly, in terms of patient care and adherence to RDT results, the key to making this strategy successful would be ensuring adequate communication with the parent regarding test results. This means providing teachers with guidelines for how to advise on appropriate medication and follow-up care.

### Comparison across contexts

Results from this review show that data in the peer-reviewed literature describing the safe and correct execution of RDTs is lacking. It is assumed that formal health care workers can safely and properly execute RDTs, though the one study examining this suggested otherwise [85]. Furthermore, interviews with health care workers revealed that some do struggle with the RDT procedures, especially with steps regarding the collecting and transferring of blood samples [19]. Though the retail sector and drug shops have a reputation for delivering lower standards of care [113], the studies reviewed show that DSVs execute RDTs well [70, 114]. For laypersons, feedback, supervision, and hands-on experience improved and helped maintain safety skills and adherence to RDT procedures. Across all contexts, the steps most commonly performed incorrectly pertained to the collection and transferring of blood samples, and the documentation of test results. Neither of these steps directly relates to patient safety, though the former could compromise the accuracy of the test results.

Accurate diagnosis is critical in the management and treatment of malaria. The results of this review showed that the diagnostic accuracy of RDTs for *P. falciparum* can be high in all the considered contexts. Sensitivities as high as 100% [71], 100% [70], and 97.9% [66], and specificities as high as 99.7% [50], 98% [70], and 98.1% [59] were observed in the formal health care sector, retail sector, and community level respectively. However, several studies conducted also reported low sensitivities and specificities, which could lead to the incorrect management of febrile illness. Low sensitivities may be caused by a variety of reasons such as user errors (e.g., collecting inadequate amounts of blood, misinterpreting RDT results, etc.), comparing RDT results against an imperfect gold standard, and low parasite densities. Comparing RDTs to PCR results may also result in lower apparent sensitivities, as PCR can detect parasite DNA remaining in a patient’s blood after an infection has been cleared, or detect very low-density (submicroscopic) infections that are possibly not a relevant comparison for RDT performance [115]. Specificity may also be impacted by user errors and comparing RDT results against imperfect gold standards, but recent malaria infections, and patient factors – such as rheumatoid factor positivity [116] – may also contribute to low specificities.

When comparing adherence to RDT results across contexts, the results of this review indicate that DSVs and CHWs generally have the highest adherence to test results and appropriately prescribe antimalarials most frequently, while adherence to RDT results is more variable in the formal health care sector. A recent systematic review conducted by Kabaghe and colleagues found similar results [117]. They concluded that RDTs have a high diagnostic accuracy, and that overall compliance to test results is fair, though lower cadres of health workers (i.e., CHWs and DSVs) displayed higher rates of adherence. The overall appropriate treatment in the formal health care sector is commonly affected by high percentages of RDT-negative patients still receiving antimalarial medications [19, 37, 49, 80]. Another important factor to consider is how RDTs change the actual and perceived roles of those performing RDTs. For example, introducing RDTs in formal health facilities likely does not substantially change the role of the health care provider or expectation of the patient. But, when examining how RDTs impact the roles of CHWs and DSVs, they fundamentally alter what services can be offered to clients. For the former, RDTs greatly expand CHW services and modifies their role from providing predominantly health promotion to curative services. For the DSVs, it is more complicated. As previously discussed, individuals generally don’t go to shops for a disease diagnosis – they go to purchase a specific drug or commodity. Thus, if a patient is seeking a diagnosis, offering RDTs represent additional services that not all drug shops may offer, making some businesses more attractive to customers. Conversely, RDTs may be a low volume commodity if customers do not necessarily want a diagnosis at a drug shop, but would prefer to exclusively purchase medication instead [54]. Still, if shops are expected to treat positive clients and refer negative clients to health facilities, DSVs may be placed in a position in which they must choose between making a profit or following guidelines. Furthermore, if an RDT-negative client already suspects that their illness is malaria but are refused antimalarial medication by the shop owner, they may seek antimalarial drugs at another shop that does not test or is known to stray from treatment guidelines.

Several concerns transcend the various contexts, perhaps most notably quality assurance and long-term sustainability of RDT use. These are especially challenging when considering wide-scale implementation at the community level and in the retail sector. Predicting how RDTs will be used outside of a closely monitored research context is difficult. Far more experience has accumulated from RDT implementation in the formal health sector than other contexts. In all contexts, but particularly the community, continuous supervision and maintaining consistent supplies of RDTs are costly and logistically challenging [118]. Additionally, in the retail sector, drug shops often have no explicit link to the formal health care sector, and monitoring RDT use could require formalizing this connection or increasing regulation.

Successful introduction and scale-up of new health technologies should be supported by policy and implementation frameworks that promote correct RDT use [119] by addressing common implementation issues such as training, supplies distribution, clinical guidelines, and supervision. Inconsistent or ambiguous frameworks may lead to inappropriate use of diagnostic technologies, which could directly impact their effectiveness. In addition, use of RDTs in public health programming requires sustained financial mechanisms to protect against RDT and antimalarial stock-outs [10, 119]. In all sectors, problems with erratic availability of RDTs and antimalarials have been reported. Indeed, five of the studies noted RDT or antimalarial stock-outs affecting the care provided by formal health care facilities [40–42, 53, 80], and others involving CHWs noted RDT or medication stock-outs [60, 61, 86].

### Limitations

This review has several limitations. First, the risk of bias was not assessed for the studies included. Consequently, we have not taken into account quality of evidence in these studies. Secondly, very few studies have been conducted in schools and the retail sector. Additionally, this review did not include gray literature. This may have limited the data included in this review. For example, only one of the identified studies in the formal health care sector investigated the safety of RDT use, though relevant information may appear in unpublished reports conducted by ministries of health or non-governmental organizations. Studies were also included regardless of sampling strategy or sample size. Small studies are likely to have lower external validity than studies with larger enrollment or stronger design. Lastly, some outcomes lacked standardized criteria. Sensitivity and specificity outcomes are highly dependent on the gold standard used as a comparison method. Typically, this was microscopy, the quality of which almost certainly varied between studies. Therefore, comparisons of sensitivity and specificity between studies may be confounded by these differences. Other outcomes lacking standardized criteria included checklists evaluating RDT safety and appropriate treatment.

## Conclusions

RDTs are used safely and effectively by CHWs, though additional research should be conducted to make the same conclusions for RDT use in the formal health care sector and retail sector. RDTs have a high diagnostic accuracy across contexts, although a worrying trend of lower specificity in the retail sector needs to be examined. Adherence to RDT results is generally high, though compliance with results tends to be lower amongst RDT-negative patients treated in the formal health care sector. If these trends of lower adherence rates are extrapolated to all of sub-Saharan Africa, thousands of patients may be incorrectly diagnosed and receive inappropriate treatment with antimalarials leading to unnecessary drug use, and an increased risk of drug resistance.

The ultimate impact of RDTs on malaria case management varies between the contexts in which care is sought, but may be linked to the nature of the client-provider interaction. Multidisciplinary research should continue to explore long-term trends and strategies to maintain safety and quality of RDT use during scale-up, especially in the retail sector and community, with an appreciation for the factors that may differ in individual contexts.

## Additional files

Additional file 1: Search Syntax. Complete list of terms used in literature search. (DOCX 84 kb)

Additional file 2: Extracted Data. Complete dataset pertaining to the RDT execution, test accuracy, or adherence to test results in sub-Saharan Africa. (XLSX 66 kb)

## Abbreviations

ACT: Artemisinin-based combination therapy
CHW: Community health worker
DSV: Drug shop vendor
HMM: Home management of malaria
HRP-2: Histidine-rich protein-2
PCR: Polymerase chain reaction
pLDH: Lactate dehydrogenase
PRISMA: the Preferred Reporting Items for Systematic Reviews and Meta-Analyses
RDTs: Malaria rapid diagnostic tests
WHO: World Health Organization
