# Supplementary material for: Use of malaria RDTs in various health contexts across sub-Saharan Africa: a systematic review
Source: BMC Public Health. 2017 May 18;17:470. doi: 10.1186/s12889-017-4398-1 (PMC5437623; doi:10.1186/s12889-017-4398-1)
Supplement: Supplementary file 1 — Search Syntax. Complete list of terms used in literature search. (DOCX 84 kb) [file 12889_2017_4398_MOESM1_ESM.docx]

**Project:** Systemic Review for Context of Use of Malaria RDTs

**Purpose:** Search Syntax

**Date:** December 12, 2016

| **Database** | **Search syntax** |
| --- | --- |
| **Medline**  [**http://www.ncbi.nlm.nih..gov/pubmed/**](http://www.ncbi.nlm.nih..gov/pubmed/)  **Literature Search Restricted From Years:**  2000 - Present | (malaria[mesh] OR malaria [tiab] OR plasmodium falciparum[tiab] OR p falciparum[tiab])  **AND**  (mass screening[mesh] OR rdt[tiab] OR rapid diagnos*[tiab] OR rapid antigen test*[tiab] OR screen*[tiab])  **AND**  (“barefoot doctor”[tiab] OR community assistants[tiab] OR community based[tiab] OR community case management[tiab] OR CCM[tiab] OR community directed[tiab] OR community drug distributor[tiab] OR community health[tiab] OR “community health workers”[mesh] OR community malaria volunteer*[tiab] OR community management[tiab] OR community owned resource person*[tiab] OR “drug shop”[tiab] OR emergenc*[tiab] OR formal health workers[tiab] OR health community worker*[tiab] OR health extension worker*[tiab] OR hmm[tiab] OR home based[tiab] OR home based management of malaria[tiab] OR home health aides[tiab] OR HMM[tiab] OR home management[tiab] OR home health worker*[tiab] OR home case management[tiab] OR hospitaliz*[tiab] OR hospitalis*[tiab] OR home health aides[mesh] OR hospital*[tiab] OR hospital[mesh] OR hospitalization [mesh] OR integrated community case management[tiab] OR iCCM[tiab] OR malaria control assistant*[tiab] OR mobile health units[mesh] OR pharmacy*[tiab] OR physician*[tiab] OR primary care[tiab] OR primary health care[tiab] OR private sector[tiab] OR “retail providers”[tiab] OR “retail shop”[tiab] OR village health worker*[tiab] OR village malaria worker[tiab]) |
